# Enhancing Chicken Fillets' Shelf Life: Synergistic Effect of Whey Protein Isolate and *Ziziphora clinopodioides* Essential Oil

**DOI:** 10.1002/fsn3.70821

**Published:** 2025-09-02

**Authors:** Amirreza Hajjar Bargh, Afshin Akhondzadeh Basti, Ali Khanjari, Negin Noori

**Affiliations:** ^1^ Department of Food Hygiene, Faculty of Veterinary Medicine University of Tehran Tehran Iran

**Keywords:** Coliform, essential oil, *Pseudomonas*, psychrotrophic, soy protein isolate, *ziziphora clinopodioides*

## Abstract

Chicken filets are among the favorite and perishable proteins in the food industry. One of the impressive ways to enhance the chicken meat's shelf life is by applying suitable coatings packaging. In this study, the antimicrobial effect of whey protein isolate (WPI) coating with *Ziziphora clinopodioides* essential oil (ZEO) (0.6%, 0.8% and 1% v/v) on the microbial load, psychrotrophic bacteria, *Pseudomonas* spp., Coliform, and LAB bacteria, chemical and organoleptic properties of chicken filets were evaluated during 12 days of cold storage. The highest antimicrobial effect was observed in the sample with WPI coating containing 1% ZEO after 12 days of storage in the refrigerator. The results showed that the treated chicken filets had a slower rate of increase in microbial count compared to the control. Additionally, chicken filets coated with WPI containing 1% ZEO exhibited the lowest microbial count at the end of the storage period. Regarding chemical data, the TVB‐N value in the control sample increased from 13.45 ± 2.02 to 38.21 ± 3.05 mg MDA/100 g, whereas in the samples containing WPI and 1% ZEO, it decreased to 25.23 ± 2.97 mg MDA/100 g. The pH values in chicken filet samples with WPI coating and 1% ZEO reached the lowest value (6.03 ± 0.1) during storage, while in the control sample, the pH increased to 6.47 ± 0.05. The final peroxide value in the control sample was 0.117 ± 0.01, but in the samples with WPI coating and 1% ZEO, it decreased to 0.105 ± 0.02. The WPI coating with ZEO also has positive effects on the chemical and organoleptic properties of chicken filets. The results showed that WPI with 1% ZEO could be applied for fresh chicken filets preserving with no undesirable sensory properties.

## Graphical abstract

Whey protein isolate (WPI) coatings incorporated with *Ziziphora clinopodioides* essential oil (ZEO) were developed. The chemical characteristics of chicken filets coated with whey protein isolate (WPI) and essential oil were evaluated. The shelf life of chicken filets was extended by WPI/ZEO treatment. WPI/1% ZEO demonstrated strong antimicrobial properties.

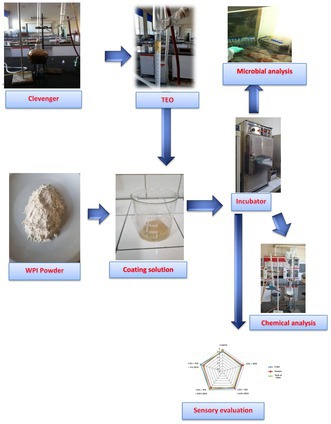

## 1 Introduction

Poultry meat is a great medium for microbial growth and spoilage (Zhu et al. 2022). Meat and poultry products have been reported to be contaminated with pathogens easily (Kumar et al. 2020). The susceptibility of poultry meat to a wide range of microbial growth can be attributed to its high water activity and nutrient content. Therefore, the meat industry must employ appropriate techniques to extend the shelf life of fresh chicken meat while preserving its nutritional quality (İncili et al. 2021; Salimiraad et al. 2022).

Nowadays, there is a growing demand for extending the shelf life of fresh chicken meat, and chemical preservatives are among the commonly used methods. However, the application of chemical preservatives in packaging poses challenges due to their potential toxicogenic, teratogenic, and carcinogenic effects. Additionally, over the past decade, increasing environmental concerns have arisen regarding the use of plastics and their detrimental impact on ecosystems (Khanjari et al. 2013). Thus, various food preservation methods have emerged, including the use of herbal essential oils, antimicrobial agents incorporated into edible films, and advanced coating technologies to enhance food shelf life (Mostafavi and Zaeim 2020). Edible coatings are thin layers applied to food surfaces. This method represents one of the most effective approaches for inhibiting microbial growth and maintaining the physicochemical properties of food products during storage (Suhag et al. 2020). Protein‐rich foods such as chicken meat can be coated with various edible materials to extend shelf life during storage (Alizadeh et al. 2019; Hassan et al. 2018; Ludwicka et al. 2020).

Essential oils (EOs) are organic volatile compounds with different mono‐ and sesquiterpenoids, phenylpropanoids, benzoids, etc. which provide antioxidant and antimicrobial properties (Omer Qader et al. 2023). EOs are considered GRAS by the USFDA (EFSA J 2010) and the antimicrobial and antioxidant activity of EOs has been evaluated in various foods (Hazrati et al. 2020; Pires et al. 2018).

*Ziziphora clinopodioides* L. (ZEO), an edible, perennial aromatic plant, belongs to the Lamiaceae family. It grows natively in several countries, including Iran, China, Turkey, Mongolia, Kazakhstan, and Kyrgyzstan. It is also known as “kakuti‐e kuhi” in Iran, “Kirnanesi” in Turkey, and “wild peppermint” in China. This plant has a long history of use as traditional medicine, like abdomen tonic, expectorant, carminative, anti‐emetic, anti‐inflammatory, and relaxing (Ding et al. 2014). The pharmacological effects of ZEO flavonoids, such as anti‐atherosclerosis properties, inducing lipid metabolism, and lessening inflammation cognate by the accumulation of inflammatory factors, through preventing neurodegenerative disorders, have been reported (Sahakyan and Petrosyan 2022; Shabbir et al. 2017).

ZEO is a potent antibacterial and antioxidant agent that is able to increase the shelf life of food products (Omer Qader et al. 2023; Esmaile et al. 2020; Shahbazi 2020; Hosseinzadeh and Ebrahimzadeh 2020; Özkan et al. 2020). Thymol and carvacrol are among the active compounds of EO with antimicrobial potency against gram‐positive and gram‐negative bacteria and fungi (Omer Qader et al. 2023).

Whey protein isolate (WPI), a nutritionally valuable milk‐derived byproduct, serves as an effective biopolymer matrix for edible coatings due to its excellent film‐forming properties and nutritional benefits (Daniloski et al. 2021). Applying WPI as a coating component would reduce the disadvantageous environmental effects on foodstuffs (Kandasamy et al. 2021; Ramos et al. 2012).

The purpose of this study was to investigate the effect of ZEO on microbial growth (total viable count [TVC], psychrotrophic bacteria, *Pseudomonas* spp., Coliform, and lactic acid bacteria [LAB]) and sensory properties of chicken filets coated with WPI during 12 days of fridge storage.

## 2 Materials and Methods

### 2.1 EO Preparation

*Z. clinopodioides* was provided from Kermanshah, Iran. The sample plant was grounded using a Lab blender and then EO was isolated by steam distillation in a Clevenger for nearly 4 h (European Pharmacopoeia 2017). Then the obtained EO was recovered and kept at 4°C for further analysis with gas chromatography–mass spectrometry (GC–MS).

### 2.2 GC–MS Analysis of EO

The GC–MS analysis of ZEO was done on a Thermo Quest Finningan apparatus (HP‐5MS 5% phenyl methylsiloxane column) and Helium (purity: 99.99%) as the carrier gas. Column temperature was initiated at 50°C and raised to 280°C at a rate of 3°C/min. The MS was set in the electron ionization mode (Shavisi et al. 2017; Sabounchi and Massoud 2016).

### 2.3 Whey Protein Isolate (WPI)

WPI was purchased from Davisco Foods International Inc. (MN, USA). Candelilla wax was obtained from Strahl and Pitsch Inc. (NY, USA). NaOH, glycerol, Rogosa, and MRS agar were provided by Merck Co. (Germany).

### 2.4 Sample Preparation

Fresh chicken filets were bought from a poultry processing industry (Boorchin, Tehran, Iran). They were put to the laboratory within 1 h on ice in polystyrene boxes. Chicken filets were cut into pieces of 60 g aseptically and dipped in the containers of whey protein isolate and different concentrations of EOs (0%, 0.6%, 0.8% and 1% v/v) for 10 min. After the marination, samples were removed and stored in a sterile bag (Interscience, France) separately at 4°C. The control and marinated samples were stored in an incubator for microbiological analyses and sensory evaluation during 12 days of fridge storage.

### 2.5 Chemical Analysis

#### 2.5.1 pH Determination

Ten grams of the chicken filet samples was mixed and homogenized in 90 mL of distilled water, and pH was measured according to Hassanin et al. (2017).

#### 2.5.2 Total Volatile Base Nitrogen (TVBN) Determination

The TVBN level was measured by the Kjeldahl method (AOAC 2012). Ten grams of sample was put in the Kjeldahl distillation system; after that, volatile nitrogen was gathered in a balloon including boric acid (2%), bromocresol green, methyl red, and then titrated with sulfuric acid (0.1 N) for measuring TVBN (mg) (AOAC 2012).

#### 2.5.3 Determination of Peroxide Value

Three grams of the sample was heated at 60°C for 3 min until the fat melted, then acetic acid and chloroform solution (3:2 v/v) were added and stirred for 3 min until the fat dissolved. After passing through a Whatman paper filter, 0.5 mL of potassium iodide solution was added and then titrated with sodium thiosulfate standard solution (Hassanin et al. 2017).

### 2.6 Microbiological Analysis

Three grams of chicken samples were placed aseptically into individual stomacher bags (Seward Medical, UK), with sterile Buffered Peptone Water (Merck) (225 mL of solution 0.1%) and homogenized in a stomacher (Bagmixer 400W, Interscience, France) for 2 min, and then serial decimal dilutions were provided in BPW solution (0.1%). After that, all developed colonies were counted and recorded (ISO 2004, 2002):

#### 2.6.1 Total Viable Count

0.1 mL of the above serial dilutions of samples was put on the surface of agar plates. TVC was monitored using plate count agar after incubating at 35°C for 2 days.

#### 2.6.2 Psychrotrophic Bacteria Count

0.1 mL of sample was added to the surface of agar plates using plate count agar after incubating at 7°C for 10 days.

#### 2.6.3 Pseudomonas Count

0.1 mL of sample was separately inoculated into duplicate Petri dishes of *Pseudomonas* selective agar with glycerol and then incubated at 25°C for 2 days.

#### 2.6.4 Coliform Count

Coliforms were enumerated with violet Red Bile Lactose agar and MacConkey agar by spreading plate method and incubated for 18 h at 35°C.

#### 2.6.5 Lactic Acid Bacteria Count

1 mL of sample was pour‐plated on MRS agar and then incubated for 2 days at 37°C.

### 2.7 Sensory Evaluation

One hundred grams of sample was cooked at 60°C for 4 min. A group of 20 panelists was applied for sensory evaluation. Panelists were requested to determine color, texture, odor, and taste of samples with a hedonic scale (0 = very bad, 9 = very good) (Fiore et al. 2021).

### 2.8 Statistical Analysis

Statistical analysis was performed by three replications on a randomized complete block design and SPSS Statistic 24 software (SPSS Inc., Hong Kong). One‐way ANOVA analysis of variance was applied at a 95% confidence level for evaluating whether the difference among factors was significant. Differences between samples were regarded as significant at *p* < 0.05.

## 3 Results and Discussion

### 3.1 Chemical Composition of ZEO

The extraction yields for ZEO was reported as 1.06% (w/w). The results of GC–MS indicated that the major components for ZEO were thymol (30.70%), p‐cymene (7.95%), borneol (12.80%), and carvacrol (7.53%). The amounts of chemical compositions and retention indices of ZEO are given in Table 1. In another study, the main constituents of ZEO were carvacrol (66.20%), thymol (20.52%), p‐cymene (5.88%), and Ç‐terpinene (5.33%) (Shahbazi et al. 2018). The chemical combinations of the EOs in spices and plants are different due to the various geographical conditions, drying methods, and the plant growth phases (Maurya et al. 2021). The quantified concentrations of carvacrol and thymol in the essential oil (EO) align with previous reports identifying these phenolic monoterpenes as the predominant bioactive constituents of ZEO (Shahbazi 2020; Alighazi et al. 2020; Shavisi et al. 2017).

**TABLE 1:** Essential oil composition of *Ziziphora clinopodioides* by GC–MS.

No	Compound	Composition (%)	Retention time (min)	Kovats index
1	Thujene	0.53	10.32	952
2	A‐Pinene	2.27	10.52	964
3	Camphene	2.92	10.81	977
4	Octen‐3‐OL	0.37	11.07	989
5	Myrecene	1.44	11.45	1007
6	A‐Terpinne	2.41	12.06	1037
7	P‐Cymene	7.95	12.14	1041
8	1,8‐Cineol	3.06	12.31	1049
9	Terpinene	6.21	12.83	1052
10	Terpinolene	1.78	13.38	1101
11	Borneol	12.80	14.76	1173
12	Menthol	1.49	15.36	1204
13	Thymol, methy ether	3.19	15.72	1225
14	Pulegone	5.3	16.3	1259
15	Thymol	30.70	16.64	1279
16	Carvacrol	7.53	16.93	1296
17	Bornyl acetate	0.93	17	1300
18	Pipritenone	3.35	17.51	1329
19	Carvacrol acetate	1.55	17.56	1332
20	B‐Bisabolene	1.9	20.08	1484
21	A‐Bisabolene	1.16	20.48	1510
22	Spathulenol	0.53	21.09	1551

### 3.2 Chemical Analysis

#### 3.2.1 pH Determination

The pH values in chicken filet samples coated with WPI containing various concentrations of ZEO (0%, 0.6%, 0.8% and 1%) during refrigeration are presented in Table 2. According to the results, it was observed that the pH levels in chicken filet samples covered with WPI and 0.6%, 0.8%, and 1% ZEO were not significantly different (*p* ≥ 0.05) during storage. The lowest pH (6.03 ± 0.1) was observed in the sample coated with WPI + 1% ZEO and the highest level (6.47 ± 0.05) belongs to the control sample at the end of storage time. It would represent that the combination of WPI+ ZEO is able to prevent the decreasing pH levels in chicken filet samples compared to the control which would be due to the effect of the film and antibacterial effect of ZEO (Omer Qader et al. 2023; Seydim and Sarikus 2006). A similar finding was noted by Rezaeifar et al. (2018), who indicated that pH levels in the range of meat coated with chitosan, lemon verbena extract 1%, and lemon verbena EO 0.5% were lower than the control sample during storage (Rezaeifar et al. 2018). It was reported that the pH values were significantly lower in coated chitosan‐based nanocomposite containing ZnO and ZEO samples than in the uncoated filets (Mosavinia et al. 2021). Similarly to our findings, the samples in nano‐liposomal EO showed a lower amount of pH than the control samples in chicken filet during storage (Kamkar et al. 2021).

**TABLE 2:** Effect of ZEO, whey protein isolate (WPI) and their composition on pH of chicken filet samples (CFS) storing at 4°C.

Samples	0 day	3rd day	6th day	9th day	12th day
Control	6.13 ± 0.08^ab^	6.36 ± 0.06^cd^	6.76 ± 0.04^gh^	6.87 ± 0.03^h^	6.47 ± 0.05^de^
CFS + WPI	6.13 ± 0.08^ab^	6.33 ± 0.05^cd^	6.7 ± 0.14^g^	6.76 ± 0.07^gh^	6.51 ± 0.15^e^
CFS + WP + 0.6% ZEO	6.13 ± 0.08^ab^	6.26 ± 0.09^bc^	6.56 ± 0.1^ef^	6.68 ± 0.1^fg^	6.33 ± 0.09^cd^
CFS + WP + 0.8% ZEO	6.13 ± 0.08^ab^	6.25 ± 0.07^bc^	6.24 ± 0.07^bc^	6.18 ± 0.07^ab^	6.14 ± 0.07^ab^
CFS + WP + 1% ZEO	6.13 ± 0.08^ab^	6.17 ± 0.07^ab^	6.13 ± 0.06^ab^	6.1 ± 0.07^ab^	6.03 ± 0.1^a^

*Note:* Various letters shows significant difference in means (*p* < 0.05).

#### 3.2.2 Determination of Total Volatile Nitrogen (TVBN)

The results of the TVBN in chicken filet samples coated with WPI containing various concentrations of ZEO (0%, 0.6%, 0.8% and 1%) during storage at refrigerator are given in Table 3. TVBN amount reached the lowest level (25.23 ± 2.97 mg/100 g) in the sample coated with WPI + 1% ZEO and at the highest level (38.21 ± 3.05 mg/100 g) in the control. It was observed that TVBN in chicken filet samples covered with WPI containing 0.6%, 0.8%, and 1% ZEO was not significantly different (*p* ≥ 0.05) during storage. It is observed that the composition of WPI + ZEO resulted in a lower increase in TVBN in chicken filet samples compared with the control sample, which is due to the protective effect of WPI film and ZEO (Hassanin et al. 2017; Kamkar et al. 2021; Rezaeifar et al. 2018). Current findings are in line with the research of Sheerzad et al. (2024) reported that coating chicken meat with WPI, nanochitosan, and 1.5% cinnamon EO resulted in significantly lower values of TVB‐N in comparison with the control group under refrigerated conditions (Sheerzad et al. 2024). Similar findings have been reported in previous studies by Wang et al. (2021) coating chicken breast filet with nanoemulsions containing 2.0% (w/v) cinnamon EO resulted in significantly lower values of TVB‐N in comparison with the control group under refrigerated conditions (Wang et al. 2021). Consistent with the findings of Farsanipour et al. (2020), fish filets coated with chitosan‐essential oil (chitosan + EO) and chitosan/whey protein isolate‐essential oil (chitosan/WPI + EO) composites exhibited significantly lower total volatile basic nitrogen (TVB‐N) values compared to control samples throughout storage. The authors attributed this phenomenon to the potent antibacterial properties of the incorporated essential oils in the active coatings (Farsanipour et al. 2020).

**TABLE 3:** Effect of ZEO, whey protein isolate (WPI) and their combination on TVN of fresh chicken filet samples (CFS) storing at 4°C.

Samples	0 day	3rd day	6th day	9th day	12th day
Control	13.45 ± 2.02^a^	18.42 ± 2.82^b^	26.94 ± 1.55^def^	33.63 ± 2.62^hi^	38.21 ± 3.05^j^
CFS + WPI	13.45 ± 2.02^a^	17.29 ± 1.91^ab^	28.41 ± 2.76^efg^	34.82 ± 2.35^ij^	38.10 ± 3.14^j^
CFS + WP + 0.6% ZEO	13.45 ± 2.02^a^	18.41 ± 2.85^b^	26.74 ± 2.29^def^	31.53 ± 1.58^ghi^	32.07 ± 2.11^ghi^
CFS + WP + 0.8% ZEO	13.45 ± 2.02^a^	17.35 ± 1.79^ab^	26.52 ± 1.95^def^	25.5 ± 2.6^de^	30.24 ± 1.12^fgh^
CFS + WP + 1% ZEO	13.45 ± 2.02^a^	17.6 ± 1.36^ab^	20.35 ± 2.14^bc^	22.85 ± 1.41^cd^	25.23 ± 2.97^de^

*Note:* Various letters shows significant difference in means (*p* < 0.05).

#### 3.2.3 Determination of Peroxide Value

The results of the peroxide value in chicken filet samples coated with whey protein isolate containing various concentrations of ZEO (0%, 0.6%, 0.8% and 1%) during refrigeration are presented in Table 4. The lowest peroxide value (0.105 ± 0.02) belongs to the sample coated with WPI + 1% ZEO, and the highest level (0.117 ± 0.01) was observed in the control sample at the end of storage. According to the results, it was observed that the peroxide value levels in chicken filet samples covered with WPI containing 0.6%, 0.8%, and 1% ZEO were not significantly different (*p* ≥ 0.05) during storage. The lower increase of peroxide value in chicken filet samples could be explained by the protective effect of WPI film and ZEO (Hassanin et al. 2017; Sheerzad et al. 2024). These findings align with Bharti et al. (2020), who demonstrated that essential oils (EOs) inhibit lipid peroxidation through two primary mechanisms: (1) scavenging lipid peroxy radicals and (2) chelating iron ions in lipoxygenase enzymes, thereby preventing oxidative degradation (Bharti et al. 2020). Based on the findings, it is concluded that herbal compounds and their derived substances, specifically CEO, are able to have a considerable influence in postponing lipid oxidation (Hussain et al. 2021; Raeisi et al. 2019, 2016; Taheri et al. 2018). The current findings corroborate those of Bazargani‐Gilani et al. (2015), demonstrating that essential oil‐based coatings serve as an effective strategy for inhibiting lipid oxidation in refrigerated chicken filets (4°C ± 1°C). Both studies confirm that active coatings containing EOs significantly (*p* < 0.05) extend the oxidative stability of poultry products during cold storage (Bazargani‐Gilani et al. 2015).

**TABLE 4:** Effect of ZEO, whey protein isolate (WPI) and their combination on peroxide value of fresh chicken filet samples (CFS) storing at 4°C.

Samples	0 day	3rd day	6th day	9th day	12th day
Control	0.061 ± 0.02^a^	0.078 ± 0.02^c^	0.095 ± 0.02^f^	0.107 ± 0.03^hi^	0.117 ± 0.01^j^
CFS + WPI	0.061 ± 0.02^a^	0.077 ± 0.01^bc^	0.094 ± 0.04^f^	0.106 ± 0.01^hi^	0.116 ± 0.03^j^
CFS + WP + 0.6% ZEO	0.061 ± 0.02^a^	0.076 ± 0.01^bc^	0.092 ± 0.03^ef^	0.105 ± 0.03^h^	0.115 ± 0.04^j^
CFS + WP + 0.8% ZEO	0.061 ± 0.02^a^	0.076 ± 0.03^bc^	0.091 ± 0.02^de^	0.099 ± 0.02^g^	0.108 ± 0.02^i^
CFS + WP + 1% ZEO	0.061 ± 0.02^a^	0.075 ± 0.02^b^	0.089 ± 0.04^d^	0.098 ± 0.02^g^	0.105 ± 0.02^h^

*Note:* Various letters shows significant difference in means (*p* < 0.05).

### 3.3 Microbiological Changes in Chicken Filets‐Shelf Life Study

#### 3.3.1 Total Viable Count (TVC)

The results of the TVC in chicken filet samples coated with WPI containing various concentrations of ZEO (0%, 0.6%, 0.8% and 1%) during storage at refrigeration are given in Table 5.

**TABLE 5:** Effect of ZEO, whey protein isolate (WPI) and their combination on TVC of chicken filet samples (CFS) storing at 4°C.

Samples	0 day	3rd day	6th day	9th day	12th day
Control	3.29 ± 0.14^a^	5.52 ± 0.25^d^	6.65 ± 0.25^f^	7.49 ± 0.19^j^	8.43 ± 0.14^k^
CFS + WPI	3.29 ± 0.14^a^	5.63 ± 0.1^d^	6.7 ± 0.16^f^	7.39 ± 0.17^hij^	8.38 ± 0.21^k^
CFS + WP + 0.6% ZEO	3.29 ± 0.14^a^	5.21 ± 0.13^c^	5.75 ± 0.16^d^	7.17 ± 0.07^ghi^	7.36 ± 0.3^ij^
CFS + WP + 0.8% ZEO	3.29 ± 0.14^a^	4.94 ± 0.06^bc^	5.55 ± 0.12^d^	7.07 ± 0.07^g^	7.31 ± 0.08^ghij^
CFS + WP + 1% ZEO	3.29 ± 0.14^a^	4.72 ± 0.16^b^	5.07 ± 0.18^c^	6.25 ± 0.13^e^	7.12 ± 0.32^gh^

*Note:* Various letters shows significant difference in means (*p* < 0.05).

As Table 5 shows, TVC in all samples of chicken filet coated with whey protein isolate with various concentrations of ZEO increased significantly (*p* < 0.05) during storage. TVC amount reached the lowest level (7.12 ± 0.32 log cfu/g) in the sample coated with WPI + 1% ZEO and the highest level (8.43 ± 0.14 log cfu/g) in the control. WPI and ZEO separately and in combination had a significant effect in controlling microbial growth (*p* < 0.05). In this research, TVC in the samples containing ZEO was lower than the control sample. Thus, the microbiological shelf life extension using either the WPI/ZEO combination was observed.

In accordance with our findings, it was observed that 0.3% ZEO used for chicken meatball had a high effect on the TVC (*p* < 0.05), showed better growth inhibitory effect of both Gram‐positive and gram‐negative bacteria (Shahbazi et al. 2017). Also, the TVC in raw beef was reduced significantly by adding the ZEO (0.1% and 0.2%) in comparison to the control sample (Shahbazi et al. 2016). Shavisi et al. (2017) confirmed that adding ZEO decreased TVC compared to the control group and extended the shelf life of fresh minced beef during 11 days of refrigeration. It was reported that a significant decrease was observed in TVC and the microbial flora in raw chicken meat treated with oregano EO (Petrou et al. 2012). Also, adding pomegranate peel extract to raw chicken meat increased the shelf life for 2 weeks during cold storage (Kanatt et al. 2010). Consistent with these findings, Ajorloo et al. (2021) observed significantly lower (*p* < 0.05) total viable counts (TVC) in fresh sausage samples treated with 0.3% Ziziphora clinopodioides essential oil (ZEO) combined with lysozyme, compared to untreated controls. This synergistic antimicrobial effect demonstrates the potential of combined natural preservatives in meat products (Ajorloo et al. 2021). The current findings align with Zhang et al. (2019), who reported significantly lower (*p* < 0.05) total viable counts (TVC) in samples treated with 0.1% and 0.5% cinnamon essential oil (CEO) compared to untreated controls. This dose‐dependent antimicrobial effect further supports the potential of CEO as a natural preservative in food applications (Zhang et al. 2019).

#### 3.3.2 Psychrotrophic Bacteria Count

The psychrotrophic counts have been applied as the important index for potential shelf life in chicken meat (Rezaloo et al. 2022). Psychotropic bacteria are among the important spoilage bacteria in meat that are able to grow under 7°C (Emiroglu et al. 2010). Psychrotrophic bacteria in chicken filet samples covered with WPI with various concentrations of ZEO increased significantly (*p* < 0.05) during cold storage (Table 6). The psychrotrophic bacteria amount was at the lowest level (6.22 ± 0.23 log cfu/g) in the sample coated with WPI + 1% ZEO and at the highest level (8.92 ± 0.08 log cfu/g) in the control. WPI and ZEO, individually or in combination, had a significant influence (*p* < 0.05) in controlling psychrotrophic bacteria growth. Therefore, the samples' shelf life was extended during storage using either WPI or the WPI/ZEO combination.

**TABLE 6:** Effect of ZEO, whey protein isolate (WPI) and their combination on psychrotrophic bacteria of chicken filet samples (CFS) storing at 4°C.

Samples	0 day	3rd day	6th day	9th day	12th day
Control	3.76 ± 0.25^a^	4.57 ± 0.1^bc^	6.77 ± 0.1^fg^	7.82 ± 0.17^h^	8.92 ± 0.08^i^
CFS + WPI	3.76 ± 0.26^a^	4.44 ± 0.12^b^	6.59 ± 0.13^ef^	7.50 ± 0.21^h^	8.12 ± 0.14^i^
CFS + WP + 0.6% ZEO	3.76 ± 0.27^a^	4.29 ± 0.05^b^	5.33 ± 0.06^d^	6.5 ± 0.8^ef^	7.13 ± 0.13^h^
CFS + WP + 0.8% ZEO	3.76 ± 0.28^a^	4.16 ± 0.14^ab^	4.86 ± 0.19^c^	5.67 ± 0.11^d^	6.69 ± 0.17^f^
CFS + WP + 1% ZEO	3.76 ± 0.29^a^	3.84 ± 0.23^a^	4.48 ± 0.18^bc^	5.63 ± 0.15^d^	6.22 ± 0.23^e^

*Note:* Various letters shows significant difference in means (*p* < 0.05).

Like TVC, all treated samples indicated significant control of psychrotrophic bacteria by ZEO. According to this study, significant differences in the psychrotrophic bacteria number of the samples with ZEO during storage time were observed (*p* < 0.05). Some previous studies stated that ZEO effectively inhibited the psychrotrophic bacteria growth in meat and fishery products (Kakaei and Shahbazi 2016; Mohebi and Shahbazi 2017; Shavisi et al. 2017). Similar results were noted by Sirocchi et al. (2017), who found that significantly lower (*p* < 0.05) Pseudomonas spp. (PSB) counts in beef samples treated with modified atmosphere packaging (MAP: 50% O_2_ + 30% CO_2_ + 20% N_2_) combined with *Rosmarinus officinalis* L. EO, compared to control samples (Sirocchi et al. 2017). Da Silveira et al. (2014) reported that fresh sausage samples treated with bay leaf essential oil (BLEO) exhibited significantly lower (*p* < 0.05) *Pseudomonas* spp. (PSB) counts compared to untreated controls during storage, with the exception of Days 0 and 4, when no significant differences (*p* > 0.05) were observed (Da Silveira et al. 2014). In a related study, Ajorloo et al. (2021) reported that the PSB count in the fresh sausage decreased significantly (*p* < 0.05) in treated samples compared with the control sample (Ajorloo et al. 2021).

#### 3.3.3 Pseudomonas Count

*Pseudomonas* spp. is among the spoilage microorganisms in meat products in cold storage (Sheir et al. 2020). Phospholipase and lipase produced by *Pseudomonas* spp. are able to degrade EOs in fresh meat and its products since having lipids and proteins rapidly at refrigerator and releasing short‐chain fatty acids that are responsible for oxidation (Darwish et al. 2024). Table 7 represented that Pseudomonas count in all samples coated with WPI with various concentrations of ZEO increased significantly (*p* < 0.05) during storage. Pseudomonas count reached the lowest level (5.14 ± 0.16 log cfu/g) in the sample coated with WPI + 1% ZEO and the highest level (8.13 ± 0.1 log cfu/g) in the control (Table 7). Therefore, WPI and ZEO combination individually and in combination had a significant effect (*p* < 0.05) in controlling microbial growth.

**TABLE 7:** Effect of ZEO, whey protein isolate (WPI) and their combination on *Pseudomonas* count of chicken filet samples (CFS) storing at 4°C.

Samples	0 day	3rd day	6th day	9th day	12th day
Control	3.63 ± 0.2^a^	4.73 ± 0.12^def^	5.66 ± 0.08^i^	7.13 ± 0.12^l^	8.13 ± 0.1^n^
CFS + WPI	3.63 ± 0.2^a^	4.67 ± 0.16^de^	5.76 ± 0.12^i^	7.02 ± 0.1^l^	8.01 ± 0.11^n^
CFS + WP + 0.6% ZEO	3.63 ± 0.2^a^	4.33 ± 0.09^bc^	5.36 ± 0.11^h^	6.85 ± 0.11^k^	7.58 ± 0.18^m^
CFS + WP + 0.8% ZEO	3.63 ± 0.2^a^	4.18 ± 0.1^b^	4.97 ± 0.15^fg^	5.34 ± 0.14^h^	6.36 ± 0.11^j^
CFS + WP + 1% ZEO	3.63 ± 0.2^a^	4.1 ± 0.12^b^	4.53 ± 0.13^cd^	4.79 ± 0.19^ef^	5.14 ± 0.16^gh^

*Note:* Different lower case superscript letters show significant differences (*p* < 0.05) in the same column.

In accordance with our findings, the initial count of *Pseudomonas* spp. in the meatball samples containing 0.1%, 0.2%, and 0.3% ZEO was decreased to 6.01, 5.12, and 4.24 log CFU/g, respectively (Shahbazi 2017). The oregano EO (1%) along with WPI film was reported to be effective against *Pseudomonas* spp. and *E. coli* in beef meat pieces (Oussallah et al. 2004). The current findings align with those of Sheerzad et al. (2024), who demonstrated that chicken meat coated with a composite of whey protein isolate (WPI), nanochitosan (NC), and 1.5% cinnamon EO (CEO) exhibited significantly lower (*p* < 0.05) *Pseudomonas* spp. (PSE) counts compared to uncoated controls during refrigerated storage (4°C ± 1°C) (Sheerzad et al. 2024). The findings of Hosseini et al. (2021) present investigation are likewise in overall alignment, as they noted that chicken meat coating with sodium alginate containing clove EO led to a reduction in PSE in the comparison control treatment (Hosseini et al. 2021). Similar results were noted by Shavisi et al. (2017) who found that the film containing minced beef with chitosan and containing 2% ZEO, 2% PE (propolis ethanolic extract) and 1% cellulose nanoparticle led to a considerable reduction in PB during refrigerated storage (Shavisi et al. 2017). A similar finding was reported by Kakaei and Shahbazi (2016), who noted that PSE were lower in chitosan‐gelatin film incorporated 2% and 2% ethanolic red grape seed extract and ZEO treatment in comparison with control (Kakaei and Shahbazi 2016).

#### 3.3.4 Coliform Count

Table 8 represented that the Coliform count in samples coated with whey protein isolate with various concentrations of ZEO increased significantly (*p* < 0.05) during storage. The Coliform count reached the lowest level (4.67 ± 0.11 log cfu/g) in the sample coated with WPI + 1% ZEO and the highest level (5.61 ± 0.12 log cfu/g) in the control. Therefore, WPI and ZEO combination individually and in combination, had a significant effect (*p* < 0.05) in controlling microbial growth.

**TABLE 8:** Effect of ZEO, whey protein isolate (WPI) and their combination on Coliform count of chicken filet samples (CFS) storing at 4°C.

Samples	0 day	3rd day	6th day	9th day	12th day
Control	2.17 ± 0.12^a^	3.12 ± 0.08^c^	3.89 ± 0.19^f^	4.66 ± 0.15^h^	5.61 ± 0.12^j^
CFS + WPI	2.17 ± 0.12^a^	3.1 ± 0.11^c^	3.81 ± 0.2e^f^	4.73 ± 0.12^h^	5.59 ± 0.13^j^
CFS + WP + 0.6% ZEO	2.17 ± 0.12^a^	2.86 ± 0.18^b^	3.64 ± 0.1^e^	4.63 ± 0.16^h^	5.50 ± 0.16^j^
CFS + WP + 0.8% ZEO	2.17 ± 0.12^a^	2.83 ± 0.12^b^	3.37 ± 0.11^d^	4.14 ± 0.09^g^	5.05 ± 0.09^i^
CFS + WP + 1% ZEO	2.17 ± 0.12^a^	2.74 ± 0.1^b^	3.28 ± 0.12^cd^	3.94 ± 0.08^fg^	4.67 ± 0.11^h^

*Note:* Different lower case superscript letters show significant differences (*p* < 0.05) in the same column.

A decrease in Enterobacteriaceae family was reported in meatball samples containing 0.3% ZEO. In some other research, ZEO was reported to present antibacterial activity against the growth of the coliform family in chicken (Shavisi 2017) and fish (Mohebi and Shahbazi 2017; Kakaei and Shahbazi 2016). The ZEO is able to reduce Enterobacteriaceae growth nearly 1–3 log CFU/g compared to control samples; adding various levels of ZEO (0.1%, 0.2%, and 0.3% vol/wt) caused an extension in the shelf life of meatball samples for minimum of 12 days (Khanzadi et al. 2020). Also, oregano essential oil (1%) along with a whey protein isolate film was reported to be effective against *Pseudomonas* spp. and *E. coli* in beef meat samples (Oussallah et al. 2004). *E. coli* in the breast chicken filets pretreated with ZEO was noticeably reduced about 4 log CFU/g (Sahebkar et al. 2020). In another study, Zhang et al. (2019) specified that the addition of 0.1% or 0.5% cinnamon essential oil substantially reduced the counts of Enterobacteriaceae during 10‐day storage, and likewise, increasing EO concentration led to a more reduction effect on Enterobacteriaceae count (Zhang et al. 2019). Maximum Enterobacteriaceae counts were found in the control group. In the study of Abbasi et al. (2021), who worked on the microbiological quality of chicken meat by corn starch coating combined with Zataria multiflora EO and CEO at refrigerated temperatures, a range of 4.30 log CFU/g to 10.94 log CFU/g was found in control samples during storage (20 days). In addition, their results determined that the integration of CEO into the coating solution reduced the number of Enterobacteriaceae by around 2.61 log CFU/g on the ultimate day of storage in comparison with the control samples (Abbasi et al. 2021). Moreover, the results of Sani et al. (2017), who investigated nanocomposite films based on cellulose nanofiber and WPI, involving titanium dioxide and rosemary EO, displayed a momentous shrink in Enterobacteriaceae counts of the wrapped lamb related to the control group, which is in edge with our outcomes (Sani et al. 2017). Researchers have described similar summons hinting that various EOs and extracts could impede the growth of the Enterobacteriaceae population in refrigerated meat and meat products (Kim et al. 2013; Moroney et al. 2012).

#### 3.3.5 LAB Count

LAB in chicken filet samples coated with whey protein isolate with various concentrations of ZEO increased significantly (*p* < 0.05) during cold storage (Table 9). LAB count was at the lowest level (7.25 ± 0.12 log cfu/g) in the sample coated with WPI + 1% ZEO and at the highest level (8.59 ± 0.22 log cfu/g) in the control (Table 9). WPI and ZEO individually and in combination had a significant effect (*p* < 0.05) in controlling LAB count growth. Therefore, the samples' shelf life extended during storage using either WPI or the WPI/ZEO combination.

**TABLE 9:** Effect of ZEO, whey protein isolate (WPI) and their combination on LAB count of chicken filet samples (CFS) storing at 4°C.

Samples	0 day	3rd day	6th day	9th day	12th day
Control	3.73 ± 0.1^a^	4.81 ± 0.15^bc^	5.85 ± 0.15^d^	7.79 ± 0.14^h^	8.59 ± 0.22^i^
CFS + WPI	3.73 ± 0.1^a^	4.86 ± 0.19^c^	5.84 ± 0.14^d^	7.67 ± 0.18^h^	8.55 ± 0.2^i^
CFS + WP + 0.6% ZEO	3.73 ± 0.1^a^	4.64 ± 0.21^bc^	5.83 ± 0.1^d^	7.6 ± 0.2^gh^	8.35 ± 0.15^i^
CFS + WP + 0.8% ZEO	3.73 ± 0.1^a^	4.56 ± 0.08^b^	5.74 ± 0.11^d^	7.35 ± 0.12^fg^	7.54 ± 0.17^gh^
CFS + WP + 1% ZEO	3.73 ± 0.1^a^	4.63 ± 0.11^bc^	5.62 ± 0.22^d^	6.79 ± 0.23^e^	7.25 ± 0.12^f^

*Note:* Different lower case superscript letters show significant differences (*p* < 0.05) in the same column.

It was reported that applying oregano EO (2%) in WPI film showed inhibitory activity against *L. plantarum* , *S. aureus* , *L. monocytogenes* , and *S. enteritidis* . It is concluded that the active combination of the EO extracts and the film material had a major effect on the antibacterial activity (Seydim and Sarikus 2006). Lactobacillus spp. was decreased during 10‐day storage period by ZEO (3%) (Shahbazi et al. 2017). It is reported that *B. subtilis* was decreased significantly in the presence of ZEO, which could be explained by the antibacterial activity of the ZEO that would associate with thymol. It represented the inhibition zones against tested bacteria, and also thymol caused the cell membranes disintegration (Kachur and Suntres 2020).

It is stated that the inhibited growth of microorganisms would be due to OH groups (in ortho and meta positions in phenolic ring) of thymol that play the role of a proton exchanger and disturb the membrane and eventually cause bacterial cell death (Shahbazi 2015). Also, the antibacterial activity of ZEO would be confirmed by the synergistic effects of lipophilic compositions like p‐cymene, a‐pinene, caryophyllene, and Ç‐terpinene that can damage cell membrane integrity, destroy cytoplasmic membrane function, and cause microorganisms death (Gyawali and Ibrahim 2014).

The volatile terpenes thymol, carvacrol, g‐terpinene, and p‐cymene showed the antimicrobial activity of ZEO. The phenolic compounds would cause protein denaturation at high concentrations. Also, the phenolic compounds would interact with membrane functions like nutrient uptake, electron transport, protein synthesis, and interfere with the proteins membrane that lead to deformation (Lobiuc et al. 2023; Marchiosi et al. 2020).

It was reported that oregano EO (2%) with WPI film showed inhibitory activity against *L. plantarum* , *E. coli O157:H7*, *L. monocytogenes* , *S. aureus* , and *S. enteritidis* . It is stated that the amount of active combinations in plant extracts and the film material had a significant effect on the biological activity of edible films (Shao et al. 2020). The coating would act as a carrier for antimicrobial composition to help in maintaining the high levels of preservatives on the surface of food products (Seydim and Sarikus 2006). The results of the present study are in general agreement with those of Bazargani‐Gilani et al. (2015) who reported a significant reduction in LAB of chicken meat dipping in pomegranate juice and coating with chitosan enriched with Zataria multiflora Boiss EO (Bazargani‐Gilani et al. 2015). In the study carried out by Da Silveira et al. (2014) it was illustrated that LAB count by adding bay leaf EO (0.05% and 0.1%) to fresh Tuscan sausage led to a significant decrease in LAB till Day 6 of storage (Da Silveira et al. 2014).

### 3.4 Sensory Evaluation

**FIGURE 1:**
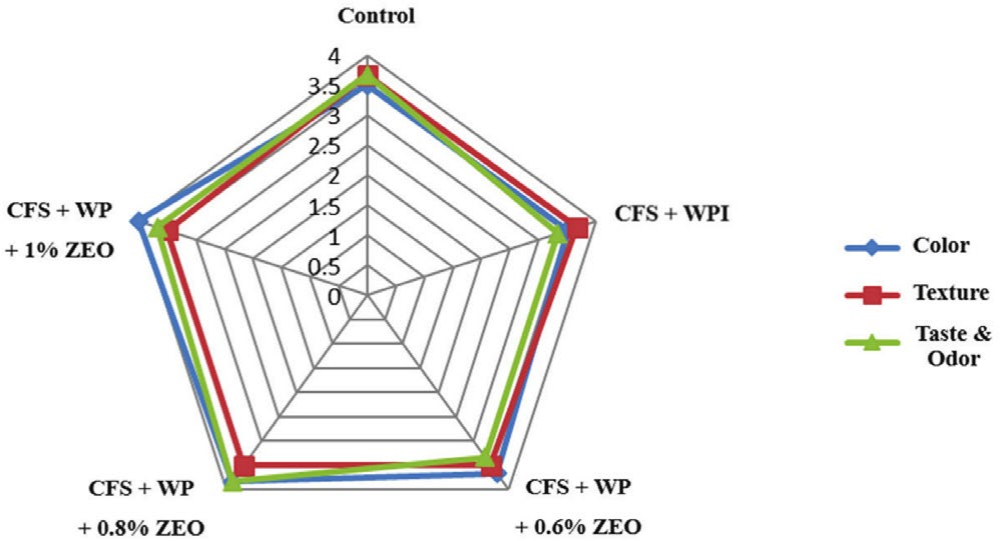
Effect of ZEO, whey protein isolate (WPI), and their combination on sensory evaluation of chicken filet samples (CFS) storing at 4°C.

The sensory evaluation (texture, taste, color and odor) of treated and control samples during fridge storage is presented in Figure 1. It can be seen that the treatments on chicken filets samples were not statistically significant (*p* ≥ 0.05). Similar results were reported by Sheerzad et al. (2024), who found that coating chicken meat with WPI, nanochitosan, and 1.5% cinnamon EO yielded the best organoleptic characteristics by the end of the storage period. The conclusions of the present study align with those of Zheng et al. (2023), who demonstrated that chitosan coatings incorporated with 1% or 2% oregano EO significantly enhanced sensory properties and extended the shelf life of chicken meat by 9 days compared to the control (Zheng et al. 2023). The present study on sensory evaluation of EO‐containing films agrees with the findings of Javaherzadeh et al. (2020) and Hematizad et al. (2021).

## 4 Conclusions

Adding EOs directly to food leads to decreasing bacterial growth. The incorporation of EOs into edible films would have suitable usage in the food industry. Using ZEO with WPI is a very promising packaging technique. The biologically active compounds of ZEO were effective in WPI films based on their bacterial inhibitory effect.

Chicken filets coated with WPI films containing ZEO had an inhibitory effect toward the microorganisms. As the amount of ZEO in WPI films was enhanced from 0.6% to 1%, a better inhibitory effect was observed against microorganisms (*p* < 0.05). At 1% of ZEO, the best inhibitory effect against microorganisms was observed.

In accordance with the findings of our study, adding various concentrations of ZEO (0.6%, 0.8% and 1%) enhances the shelf life of chicken filets effectively for 12 days and led to a decrease in the TVB‐N level and PV values in comparison with uncoated samples. Additionally, the TVC and the growth of *Pseudomonas* spp., Coliform bacteria, psychrotrophic, and LAB bacteria were decreased. According to the results that indicate the positive synergistic effect of the materials used in this research, as well as the increasing demand of consumers to use natural compounds with multiple properties (antioxidant, antimicrobial, shelf‐life extender, etc.), the deployment of these types of coatings in foods, especially meat products such as chicken meat, is recommended.

It is concluded that 1% ZEO could be used to preserve the fresh chicken filets with no unfavorable sensory properties.

## Author Contributions

**Amirreza Hajjar Bargh:** writing – draft, editing, visualization, methodology, investigation, formal analysis. **Afshin Akhondzadeh Basti:** writing and reviewing, supervision, project administration, funding acquisition. **Ali Khanjari:** writing draft, editing, validation, analysis, data gathering. **Negin Noori:** editing, visualization, data gathering.

## Ethics Statement

In this work, a group of panelists was corporated for sensory evaluation and no animal testing was conducted.

## Conflicts of Interest

The authors declare no conflicts of interest.

## Data Availability

The data used to support the findings of this study are included within the article.
